# 

**Published:** 1883-10

**Authors:** 


### CONTENTS.

### EXTRACTS.

Increase of Doctors in Atlanta........ 1
The Englishman's Fireside........ 5
Anteflexion of the Uterus with Stenosis of the Cervical Canal and Dysmenorrhœa, Treated by Rapid Dilatation.—Flatulent Distention of the Stomach. 12
A Point in the Progress of Therapeutics........ 17
Cactus Grandiflorus in Sexual Exhaustion........ 19
A Peculiar Case........ 19
The Therapeutic Value of Corn Silk........ 20
Oleate of Copper for Freckles........ 20

### EDITORIAL.

Practical Chemistry for Students and Physicians—Chapter Fourth........ 21
Exercise Questions on Chapter Fourth........ 27
Transactions of the Medical Association of Georgia........ 28
Medical School of a Northern University........ 29
Medical Teachin · in Louisville, Kentucky........ 29
Old Atlanta Medical College........ 30
Dr. M. J. Ely........ 30
Tribute of Respect—Dr. M. J. Ely........ 31
Sylvester F. Mixer, M.D., of Buffalo........ 31
BOOKS........ 32
PAMPHLETS........ 32

